# Contribution of the Purinergic Receptor P2X7 to Development of Lung Immunopathology during Influenza Virus Infection

**DOI:** 10.1128/mBio.00229-17

**Published:** 2017-03-28

**Authors:** Victor H. Leyva-Grado, Megan E. Ermler, Michael Schotsaert, Ma G. Gonzalez, Virginia Gillespie, Jean K. Lim, Adolfo García-Sastre

**Affiliations:** a Department of Microbiology, Icahn School of Medicine at Mount Sinai, New York, New York, USA; b Global Health and Emerging Pathogens Institute, Icahn School of Medicine at Mount Sinai, New York, New York, USA; c Center for Comparative Medicine and Surgery, Icahn School of Medicine at Mount Sinai, New York, New York, USA; d Department of Medicine, Division of Infectious Diseases, Icahn School of Medicine at Mount Sinai, New York, New York, USA; Brown University

**Keywords:** cytokines, extracellular ATP, immunopathology, influenza, purinergic receptor

## Abstract

An exacerbated immune response is one of the main causes of influenza-induced lung damage during infection. The molecular mechanisms regulating the fate of the initial immune response to infection, either as a protective response or as detrimental immunopathology, are not well understood. The purinergic receptor P2X7 is an ionotropic nucleotide-gated ion channel receptor expressed on immune cells that has been implicated in induction and maintenance of excessive inflammation. Here, we analyze the role of this receptor in a mouse model of influenza virus infection using a receptor knockout (KO) mouse strain. Our results demonstrate that the absence of the P2X7 receptor results in a better outcome to influenza virus infection characterized by reduced weight loss and increased survival upon experimental influenza challenge compared to wild-type mice. This effect was not virus strain specific. Overall lung pathology and apoptosis were reduced in virus-infected KO mice. Production of proinflammatory cytokines and chemokines such as interleukin-10 (IL-10), gamma interferon (IFN-γ), and CC chemokine ligand 2 (CCL2) was also reduced in the lungs of the infected KO mice. Infiltration of neutrophils and depletion of CD11b^+^ macrophages, characteristic of severe influenza virus infection in mice, were lower in the KO animals. Together, these results demonstrate that activation of the P2X7 receptor is involved in the exacerbated immune response observed during influenza virus infection.

## IMPORTANCE

A hallmark of influenza virus infection is the development of lung pathology induced by an exacerbated immune response. The mechanisms shared by the antiviral host defense required for viral clearance and those required for development of immunopathology are not clearly understood. Purinergic receptors, and in particular the purinergic receptor P2X7 (P2X7r), are involved in activation of the immune response. We used mice lacking the P2X7r (P2X7r KO mice) to better understand the mechanisms that lead to development of lung pathology during influenza virus infection. In our studies, we observed that P2X7r KO mice developed less lung immunopathology and had better survival than the wild-type mice. These results implicate P2X7r in the induction of an exacerbated local immune response to influenza virus and help us to better understand the mechanisms leading to the lung immunopathology observed during severe viral infections.

## INTRODUCTION

Influenza virus infection is an acute respiratory disease that causes high morbidity and mortality and represents an important threat to public health. Activation of the host antiviral innate and adaptive immune responses typically results in self-limitation of the disease (1–3). Nevertheless, it is clear that influenza virus infection still causes high morbidity and mortality in infected individuals (4).

A hallmark in many fatal cases of influenza infection is the development of exacerbated lung immunopathology caused by an unbalanced immune response. Therefore, the response needs to reach a balance to effectively eliminate the invading virus while avoiding the development of immune-mediated pathology of the lungs (1). Although the causes of immunopathology such as excessive production of cytokines and infiltration of inflammatory cells have been reported for influenza virus infection (2, 3), the mechanisms shared by the antiviral host defense required for viral clearance and those required for development of immunopathology are not clearly understood (5, 6).

The purinergic receptor P2X7 (P2X7r) is a trimeric ligand-gated ion channel activated by extracellular ATP (7). P2X7r is constitutively expressed on many cell types, including respiratory epithelial cells and most immune cells, such as neutrophils, monocytes/macrophages, dendritic cells, natural killer cells, and B and T lymphocytes (8–11). Activation of P2X7 receptors by extracellular ATP plays a central role in the induction of inflammation (12–14). For example, it triggers the release of proinflammatory cytokines, including interleukin-1α (IL-1α), IL-1β, IL-6, IL-10, and IL-18 (14, 15) and prompts activation of NLPR3 inflammasome (12, 16, 17), production of reactive oxygen species (18–20), and induction of apoptosis (21, 22).

The role of purinergic signaling through the P2X7r during virus infection has been demonstrated using different models. During HIV infection, the activation of the pathway is important for cell-to-cell infection (23) and for the release of infectious particles from infected macrophages (24). Studies of peripheral blood mononuclear cells (PBMC) from hepatitis C virus-infected patients showed that the upregulation of the P2X7r correlates with a sustained antiviral response after treatment (25). In human PBMC infected with dengue virus, activation of the purinergic signal through P2X7r modulates the immune response by reducing virus replication and killing of infected cells by γδ T cells (26, 27). Finally, mice lacking the P2X7r (P2X7r KO mice) have increased survival to adenovirus infection compared to wild-type (WT) mice (28). Some studies suggest that activation of purinergic signaling through the P2X7r may be important for the immune response to influenza virus infection. A partial reduction in IL-1β secretion was observed in bone marrow-derived macrophages and dendritic cells from virus-infected P2X7r KO mice, indicating a possible role for the receptor in NLRP3 activation (29). In a different study using tumor necrosis factor alpha (TNF-α) receptor KO mice, it was observed that after influenza virus infection, the mRNA levels of the P2X7r were lower in these mice, suggesting a role for this pathway in the TNF-α signaling after infection (30). In addition, the levels of extracellular ATP (the P2X7r ligand) increase in the lung airways after influenza virus infection (31, 32). To determine the possible contribution of the P2X7r pathway in the induction of an exacerbated immune response during influenza virus infection, we tested the survival, cytokine production, leukocyte infiltration, and lung pathology after infection in P2X7r KO mice and compared it to wild-type mice. Overall, the KO mice have a reduced inflammatory response in the lungs, with a lower degree of lesion development, which resulted in increased survival.

## RESULTS

### Pretreatment of A549 cells with a P2X7 receptor antagonist reduces virus growth.

**FIG 1:**
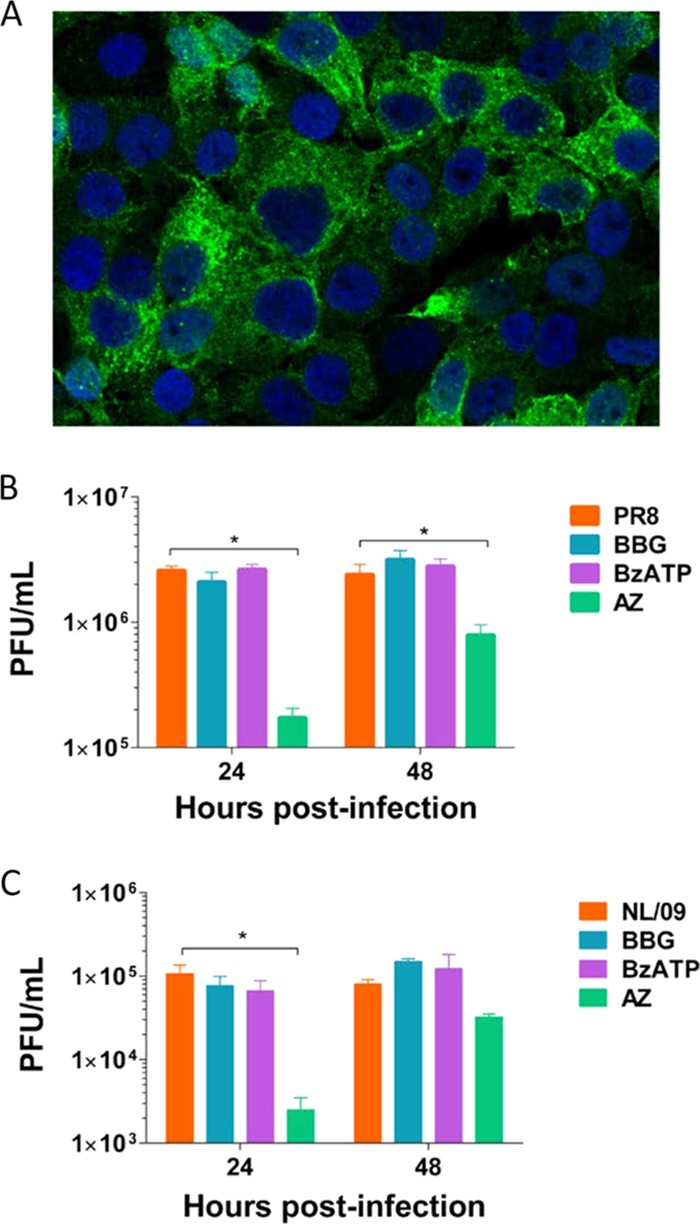
Effects of purinergic receptor P2X7 antagonists on influenza virus growth in A549 cells. (A) Uninfected A549 cells stained for the presence of the purinergic receptor P2X7 (green) and cell nuclei (blue). (B) Virus titers at 24 and 48 h postinfection of A549 cells infected with influenza A/Puerto Rico/08/1934 (PR8). Before infection, cells were incubated for 1 h with 100 µM brilliant blue G (BBG) (a purinergic receptor antagonist), 3-[1-[[(3′-nitro[1,1′-biphenyl]-4-yl)oxy]methyl]-3-(4-pyridinyl)propyl]-2,4-thiazolidinedione (AZ) (a purinergic receptor antagonist), or 2′(3′)-*O*-(4-benzoylbenzoyl)ATP tri(triethylammonium) salt (BzATP) (a purinergic receptor agonist). (C) Virus titers at 24 and 48 h postinfection of A549 cells infected with influenza A/Netherlands/602/2009 (NL/09). Before infection, cells were incubated for 1 h with 100 µM BBG, BzATP, or AZ. This figure shows the results of a representative experiment of three replicate experiments. Values that are significantly different (*P* < 0.05) are indicated by a bar and an asterisk.

To determine whether activation of the P2X7r has an effect on influenza virus growth, we tested the addition of BBG (brilliant blue G) (a P2X7r antagonist), AZ11645373 {3-[1-[[(3′-nitro[1,1′-biphenyl]-4-yl)oxy]methyl]-3-(4-pyridinyl)propyl]-2,4-thiazolidinedione} (AZ) (a P2X7r antagonist), or BzATP [2′(3′)-*O*-(4-benzoylbenzoyl)ATP triethylammonium] (a P2X7r agonist) to A549 cell cultures before infection. First, we confirmed by immunofluorescence confocal microscopy that these cells expressed the P2X7 receptor (Fig. 1A). One hour before infection with influenza A/Puerto Rico/8/1934 H1N1 (PR/8) virus or A/Netherlands/602/2009 H1N1 pandemic (H1N1pdm) (NL/09) virus, cells were treated with BBG, BzATP, or AZ11645373. No significant differences were observed in PR/8 virus growth in cells treated with BBG or BzATP compared to the untreated cells (*P* = 0.1) (Fig. 1B). We observed a significant reduction in virus titers in cells pretreated with AZ and infected with PR/8 at 24 h and 48 h postinfection compared to untreated cells (*P* < 0.05) (Fig. 1B). In cells infected with NL/09 virus and treated with BBG or BzATP, no significant differences were observed in virus growth (Fig. 1C). In cells pretreated with AZ and infected with NL/09 virus, we observed a significant reduction in virus titers at 24 h postinfection compared to untreated cells (*P* < 0.05) (Fig. 1C).

### Mice lacking P2X7 receptors have a better outcome after influenza virus infection.

**FIG 2:**
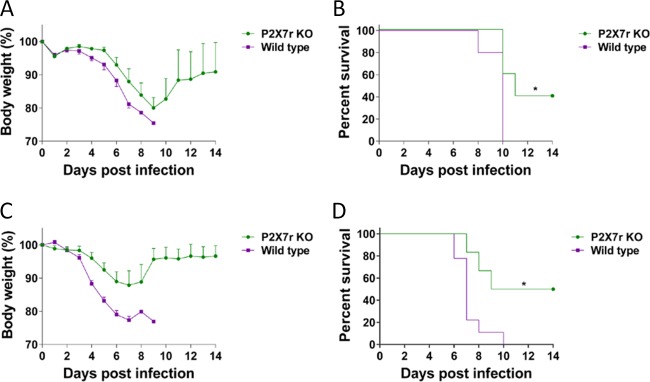
Body weight and survival curves of P2X7 receptor KO mice infected with influenza virus. (A and B) Groups of wild-type (WT) mice or mice with the P2X7 receptor knocked out (P2X7r KO mice) were infected with influenza A/Puerto Rico/08/1934 virus, and body weight (A) and survival (B) were recorded for 14 days. A significant difference (*P* < 0.05) in survival was observed in the knockout group (*). (C and D) A different set of knockout (KO) or wild-type mice were infected with influenza A/Netherlands/604/2009 H1N1pdm virus, and body weight (C) and survival (D) were recorded for 14 days. A significant difference (*P* < 0.05) in survival was observed in the knockout group (*). This figure shows the results of a representative experiment of two repetitions with six mice in each KO group and eight mice in each WT group.

Activation of the P2X7r has been associated with an increased and sustained inflammatory response. To determine whether this pathway is related to the exacerbated lung immunopathology developed after influenza virus infection, we tested mice lacking the P2X7r in our mouse model of influenza virus infection. After infection with PR/8 virus, we observed a significant reduction in body weight at days 8 and 9 postinfection in the wild-type (WT) mice (*P* < 0.05) (Fig. 2A). We also observed increased survival in the virus-infected P2X7r KO mice (40%) compared to the wild-type mice (0%) (*P* < 0.05) (Fig. 2B). Similarly, in wild-type mice infected with NL/09 virus, we observed a significant reduction in body weight at days 4 to 8 postinfection (*P* < 0.05) (Fig. 2C). A significant increase in survival was observed in the infected P2X7r KO mice (50%) compared to the infected wild-type mice (0%) (*P* < 0.05) (Fig. 2D).

### Reduced histopathology in the lungs of P2X7r KO mice infected with the NL/09 virus.

**FIG 3:**
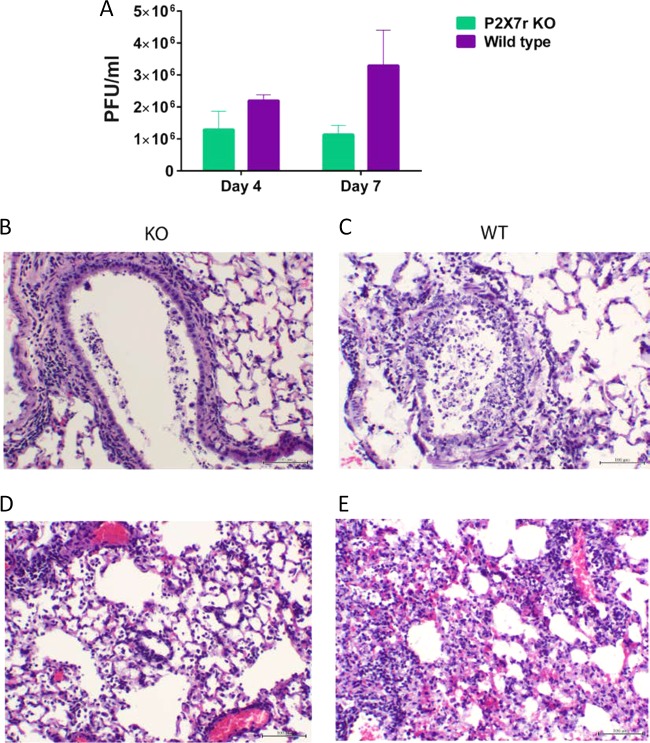
Virus lung titers and histopathology in purinergic receptor P2X7 KO mice infected with influenza A/Netherlands/604/2009 H1N1pdm virus (NL/09). (A) Lung virus titers expressed in PFU per milliliter at 4 or 7 days postinfection. From a different set of mice, the lungs were collected on day 4 postinfection, and representative sections were prepared for histopathology. (B) Bronchiolar region from a P2X7r KO mouse lung showing mild necrosis and peribronchiolar inflammation. (C) Bronchiolar region from a wild-type (WT) mouse lung with moderate to marked necrosis and moderate peribronchiolar inflammation. (D) Alveolar section from a P2X7r KO mouse lung with mild inflammation characterized by scattered cell necrosis with moderate infiltration of inflammatory cells. (E) Alveolar section from a wild-type mouse lung showing marked alveolar inflammation characterized by multifocal vacuolation with segmental necrosis and marked infiltration of inflammatory cells. This figure shows results representative of two replicate experiments with five mice in each KO group and five mice in each WT group. Bars = 100 µm.

The amount of replicating virus in the lungs is thought to correlate with the degree of lung pathology. Therefore, we infected wild-type and P2X7r KO mice with NL/09 virus, and at 4 or 7 days after infection, the lungs were harvested, and viral titers were determined by plaque assay. Reduced virus titers were observed in the lungs of the P2X7r KO mice compared to the wild-type mice, particularly in the samples collected on day 7 postinfection, although these differences were not statistically significant (*P* = 0.1) (Fig. 3A). In a different set of mice, the lungs were collected on day 4 postinfection, and representative sections were prepared for histopathology. In both groups of mice, lesions were characterized as bronchointerstitial pneumonia, although the severity of the lesions differed in the groups (*P* < 0.05) (Table 1). In the lungs of the virus-infected P2X7r KO mice, the bronchiolar region showed mild to moderate epithelial degeneration, characterized by vacuolation and multifocal necrosis of individual epithelial cells with a thin layer of peribronchiolar inflammatory cells, and moderate perivascular inflammation with little to no presence of intraluminal debris (Fig. 3B). The bronchiolar region of the lungs from wild-type mice exhibited a marked epithelial degeneration characterized by vacuolation, segmental necrosis, and loss of the epithelium, with a thick layer of peribronchiolar inflammatory cells, and mild to marked amounts of intraluminal debris (Fig. 3C). At the alveolar level, lesions in the KO mice consisted of mild inflammation as well as tissue damage that was characterized by the presence of scattered cell necrosis with mild to moderate infiltration of inflammatory cells (Fig. 3D). The alveolar region of lungs from wild-type mice exhibited marked alveolar inflammation characterized by multifocal necrosis and marked infiltration by inflammatory cells (Fig. 3E).

**TABLE 1:** Histopathology scores of lungs from P2X7 receptor knockout mice infected with influenza A/Netherlands/602/2009 virus

Group	Lung pathology score^a^					
	Amt tissue affected	Perivascular inflammation	Epithelial degeneration	Peribronchiolar inflammation	Intraluminal debris	Alveolar inflammation
P2X7r KO	1	1.6	1.6*	1.6	0.6*	1.3*
Wild-type	1.3	1.3	3	2	2.3	2.3

^a^ Lung pathology was scored on a scale of 0 to 4. For amount of tissue affected, lung pathology was scored as follows: 0, none; 1, <25% of section; 2, 25 to 50% of section; 3, 50 to 75% of section; 4, >75% of section. For inflammation and epithelial degeneration, lung pathology was scored as follows: 0, no lesions; 1, mild changes with scattered cell necrosis/vacuolation and few/scattered inflammatory cells in the bronchiolar epithelium with minimal perivascular inflammation; 2, moderate multifocal vacuolation and/or cell necrosis with peribronchiolar and/or perivascular inflammation (<5 cell layers thick); 3, marked, multifocal/segmental necrosis, epithelial loss/effacement and peribronchiolar and/or perivascular inflammation (>5 cell layers thick); 4, severe, coalescing areas of necrosis, parenchymal effacement with confluent areas of inflammation. For intraluminal debris, lung pathology was scored as follows: 0, none; 1, mild amounts; 2, moderate amounts; 3, marked amounts; 4, severe amounts. Each score value represents the mean of five mice per group. Overall pathology scores for the P2X7 KO group that are significantly different (*P* < 0.05) from those of the wild-type group are indicated by an asterisk.

### Reduction of apoptosis in lungs of mice lacking the P2X7r and infected with influenza A virus.

**FIG 4:**
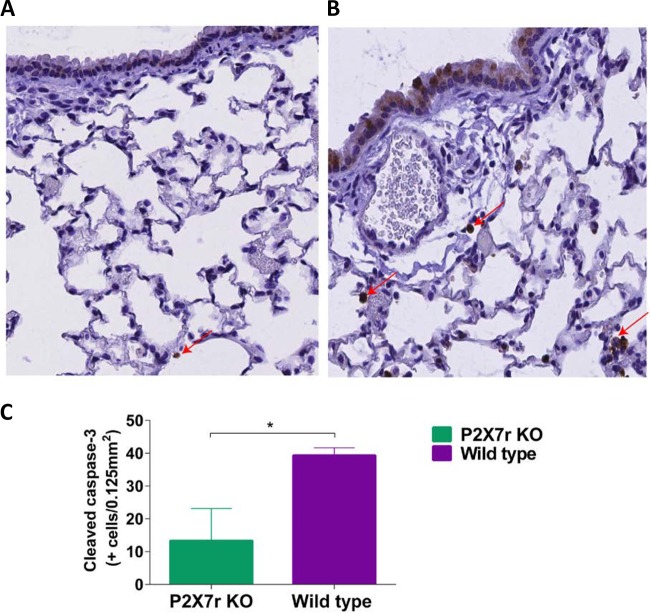
Apoptotic cells in lungs of purinergic receptor P2X7 KO mice infected with influenza A/Netherlands/604/2009 H1N1pdm virus. Lungs were harvested from each group on day 4 postinfection, and representative sections were prepared for immunohistochemistry analysis of cleaved caspase-3. (A) Alveolar section from a P2X7r KO mouse lung showing a small number of cells positive for the cleaved caspase-3 antigen (brown; indicated by red arrows). (B) Alveolar section from a wild-type mouse lung. (C) Quantification of the number of immunoreactive cleaved caspase-3 cells in the lungs of infected mice. This figure shows results of a representative experiment of two replicate experiments with five mice in the KO group and five mice in the WT group. The asterisk indicates a significant difference (*P* < 0.05).

Signaling through the P2X7 receptor induces cell apoptosis by activation of caspase cascades, including caspase-1, -3, and -8. To determine the levels of apoptosis after infection with influenza A NL/09, we quantified the number of cleaved caspase-3 immunoreactive cells in the lungs of P2X7r KO and wild-type mice collected on day 4 postinfection. Lung sections at the alveolar region showed a reduced number of cleaved caspase-3-positive cells in samples obtained from virus-infected P2X7r KO mice compared to virus-infected WT mice (Fig. 4A and B). After quantification, we confirmed that the number of cleaved caspase-3-positive cells was significantly lower in the lungs of the infected KO mice (*P* < 0.05), (Fig. 4C), indicating a reduced amount of apoptosis in the lungs of these mice.

### The production of proinflammatory cytokines is lower in the virus-infected P2X7r KO mice than in the wild-type mice.

**FIG 5:**
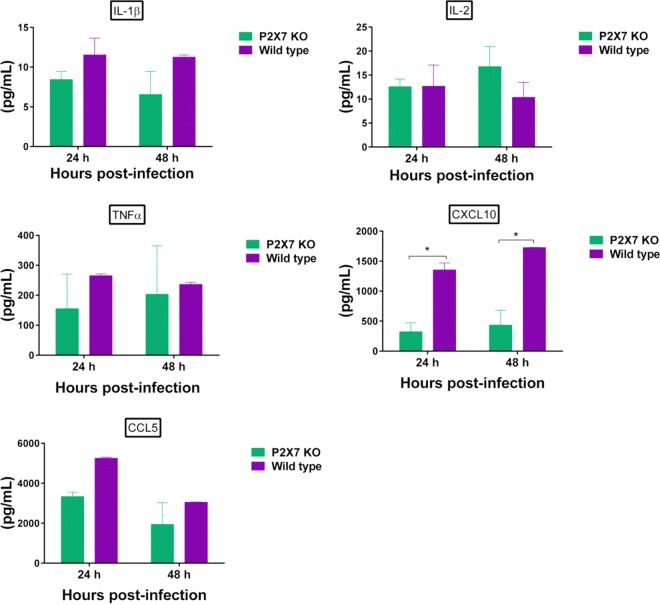
Cytokine production in mouse bone marrow-derived macrophages infected with influenza A/Netherlands/602/2009 H1N1pdm virus. Bone marrow-derived macrophage cell culture supernatants were collected and used to determine the amount of cytokine protein (in picograms/milliliter) at 24 and 48 h postinfection by an enzyme-linked immunosorbent assay (ELISA). Cytokines evaluated included interleukin-1β (IL-1β), IL-2, tumor necrosis alpha (TNF-α), CXC chemokine ligand 10 (CXCL10), and CC chemokine ligand 5 (CCL5). This figure shows the results of a representative experiment of two replicate experiments with three technical replicates per group. An asterisk indicates a significant difference (*P* < 0.05).

**FIG 6:**
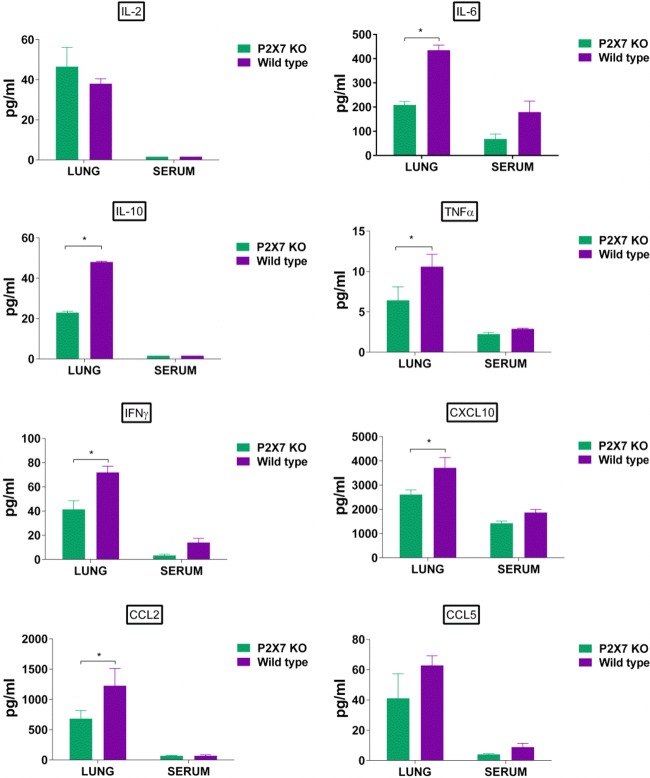
Lung cytokine production in purinergic receptor P2X7 knockout or wild-type mice infected with influenza A/Netherlands/604/2009 H1N1pdm virus. Lungs and serum samples (five mice per group) were collected on day 3 postinfection and processed for multiplex ELISA to determine the amount of cytokine protein (in picograms per milliliter) in each sample. Cytokines and chemokines evaluated included IL-2, IL-6, IL-10, TNF-α, interferon gamma (IFN-γ), CXCL10, CCL2, and CCL5. This figures shows the results of a representative experiment of two replicate experiments with five mice in each group (KO and WT). An asterisk indicates a significant difference (*P* < 0.05).

To determine whether the reduced pathology and apoptosis observed in the lungs of the P2X7r KO mice were correlated with decreased cytokine production after infection, we first quantified the amount of cytokines in *ex vivo*-infected bone marrow-derived macrophages (BMDM) and then in the lungs of both virus-infected P2X7r KO and WT mice. Cultured BMDM, obtained from P2X7r KO and WT mice, were infected with NL/09 virus, and supernatants were collected at 24 and 48 h postinfection. We observed an increased production of CXC chemokine ligand 10 (CXCL10) at both time points in the supernatant of infected macrophages obtained from the WT mice (*P* < 0.05) (Fig. 5). No statistical differences were observed for other cytokines such as IL-1β, IL-2, tumor necrosis factor alpha (TNF-α), and CC chemokine ligand 5 (CCL5), although a trend to significance was observed in the amount of CCL5 in samples collected at 24 h postinfection (*P* = 0.07). For the *in vivo* experiments, we first infected a set of mice (P2X7r KO and WT) with the influenza NL/09 virus, and then on day 3 postinfection, we collected lungs and sera. The amounts of IL-6, IL-10, TNF-α, IFN-γ, CXCL10, and CCL2 were significantly higher in the lungs of the WT mice than in the lungs of the KO mice (*P* < 0.05) (Fig. 6). A trend to significance was observed in the amount of IL-2 in the lungs of the KO mice compared to the lungs of the WT mice (*P* = 0.08) (Fig. 6). Similar results were observed in mice infected with a different influenza virus, PR/8, with significantly larger amounts of IL-10, IFN-γ, CXCL10, CCL2, and CCL5 in the lungs of the WT mice (see Fig. S1 in the supplemental material).

10.1128/mBio.00229-17.1 FIG S1  Lung cytokine production in purinergic receptor P2X7 knockout or wild-type mice infected with influenza A/Puerto Rico/08/1934 H1N1 virus. Lungs and serum samples (five mice in each group) were collected on day 3 postinfection and processed for multiplex ELISA to determine the amount of cytokine protein (in picograms per milliliter) in each sample. Cytokines and chemokines evaluated included interleukin-2 (IL-2), IL-6, IL-10, tumor necrosis alpha (TNF-α), gamma interferon (IFN-γ), CXCL10, CCL2, and CCL5. This figure shows results of a representative experiment of two replicate experiments with five mice in each group (KO and WT). An asterisk indicates a significant difference (*P* < 0.05). Download FIG S1, PDF file, 0.4 MB. Copyright © 2017 Leyva-Grado et al. 2017 Leyva-Grado et al. This content is distributed under the terms of the Creative Commons Attribution 4.0 International license.

### The percentages of pulmonary activated macrophages and activated lymphocytes were higher in the P2X7 KO mice after infection.

**FIG 7:**
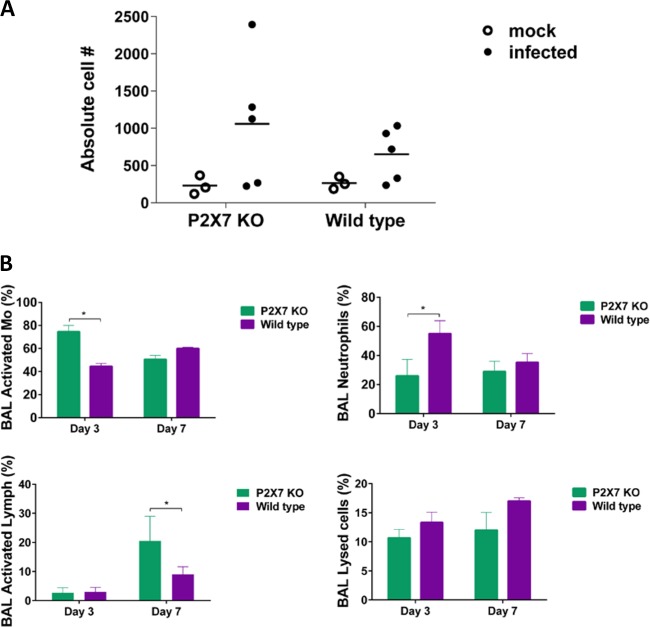
Lung leukocyte populations in purinergic receptor P2X7 knockout or wild-type mice infected with influenza A/Netherlands/604/2009H1N1pdm virus. (A) The number of CD11b^+^ macrophages isolated from enzymatically digested lungs on day 7 postinfection was determined by flow cytometry using specific cell markers. (B) Leukocytes recruited to the airway space were collected by lavaging the lungs, and then bronchoalveolar lavage (BAL) fluid was collected. The type of cells in each sample was evaluated from at least 200 cells counted under the microscope and expressed as a percentage (five mice in each group group). An asterisk indicates a significant difference (*P* < 0.05). Mo, macrophages.

We evaluated the myeloid cell population in the whole lungs of P2X7 KO and WT mice infected with NL/09 virus and collected on day 3 or day 7 postinfection (p.i.). We observed a higher number of CD11b^+^ macrophages in the lungs of virus-infected P2X7 KO mice at both time points compared to the virus-infected WT mice (*P* = 0.056) (Fig. 7A) with no significant differences in the absolute number of cells between mock-infected or virus-infected WT and P2X7 KO samples.

We then evaluated the leukocyte population in the bronchoalveolar lavage fluid (BALF) of both P2X7 KO and WT mice 3 and 7 days postinfection with NL/09 virus. The total number of cells and the viability in BALF were not different between the groups. The percentage of activated macrophages was significantly increased at day 3 p.i. in the P2X7 KO mice than in the WT mice (*P* < 0.05), but not at day 7 p.i. (Fig. 7B). The percentage of neutrophils in the BALF samples was significantly higher at day 3 p.i. in the WT mice than in the P2X7 KO mice (Fig. 7B) (*P* < 0.05). No differences between groups were observed at day 7 p.i. A significantly higher percentage of activated lymphocytes was observed in the KO mice at day 7 p.i. (*P* < 0.05) (Fig. 7B). No differences in the percentage of activated lymphocytes were observed between groups at day 3 p.i. The percentage of lysed cells in the BALF of WT mice tended to be higher, especially at day 7 p.i. (*P* = 0.1) (Fig. 7B).

## DISCUSSION

The mechanisms by which the immune response is balanced to achieve influenza virus clearance and tissue repair versus harmful immunopathology are not well understood (5, 6, 33). In this study, we showed that mice lacking the purinergic receptor P2X7 have a better clinical outcome after influenza A virus infection characterized by an increased survival rate with an overall reduced immunopathology of the lungs compared to the wild-type mice.

ATP, the main ligand for P2X7 receptor, is usually located intracellularly, although small amounts can be found in the extracellular space (10, 34, 35). When cells are under stress or damaged by infection, ATP is released into the extracellular space and rapidly binds to excitatory receptors such as P2X7 to promote an inflammatory response (9, 10, 34). Activation of the purinergic pathway through P2X7r encompasses a series of inflammation-related responses, including activation, proliferation, and recruitment of immune cells, as well as secretion of diverse inflammatory mediators, including cytokines and chemokines (7, 9, 10).

The initial evaluation was done *in vitro* by infecting A549 cells and treating them with different P2X7r agonist and antagonists before influenza virus infection to determine the impact on virus growth. We observed differences only in cells treated with the P2X7r antagonist AZ11645373. AZ is a potent cyclic imide (50% inhibitory concentration [IC_50_], 5 to 10 nM) that selectively inhibits the human P2X7r (36). In contrast, BBG, the other P2X7r antagonist used in this study, is moderately selective and less potent in blocking the human P2X7r (IC_50_, 200 nM) (37). Since we treated cells with the same concentration of compound (100 µM), we speculate that differences in potency may be one of the reasons why we observed some effect in virus growth with one receptor antagonist but not with the other.

For the *in vivo* studies, we first observed that mice lacking the P2X7 receptor (KO mice) had better survival after infection and that survival was independent of the virus strain used. It is generally accepted that survival of the infected host correlates with a reduction of the viral burden in the lung (38, 39). However, when we quantified the virus titers in the lungs of infected mice, we observed that the virus titers in the lungs of virus-infected KO mice were not significantly different from those in the lungs of virus-infected wild-type mice. This lack of correlation between lung virus titers and severity of disease has been previously reported (6, 40, 41). In early stages of viral infection, influenza A virus (IAV) activates antiapoptotic signals to evade the host defense mechanism. However, in the later stages of infection, IAV triggers apoptosis through activation of intrinsic pathways in order to achieve enhanced viral replication and dissemination (42, 43), contributing to tissue damage. We evaluated apoptosis in lungs at day 4 postinfection in what can be considered late in infection, and we observed reduced numbers of apoptotic cells in the KO mice. Apoptosis can occur through many mechanisms, including the production of proinflammatory cytokines leading to an increased burden of apoptosis of the alveolar epithelium (44), activation of caspase cascades, including caspase-1, -3, and -8 (45), and the formation and release of reactive oxygen species (46). It is interesting that activation of the P2X7 receptor induces some of the aforementioned proapoptotic mechanisms (45, 46); therefore, the absence of this receptor is causing a reduction in apoptosis during later stages of infection.

The extent and severe damage observed in the lung tissues of the virus-infected WT mice can be due to both the cytolytic effect of the virus infection itself and to immunopathology caused by immune cells targeting infected cells. We did not find a significant difference in lung virus titers between the virus-infected KO and WT mice; therefore, the reduced histopathology and apoptosis observed in the KO mice pointed toward differences in immune-mediated pathology.

During influenza virus infection, an increased production of proinflammatory cytokines positively correlates with severity of disease in humans, especially during infection with highly virulent strains of the virus (38, 47, 48). During the recent H1N1 pandemic, patients with severe disease also had high levels of proinflammatory cytokines (39). A similar correlation has been observed in animal models of influenza virus infection (49, 50). In the mouse model of influenza, mice with severe clinical signs that succumb to infection have increased levels of proinflammatory cytokines and chemokines in the lungs and develop lung immunopathology (50–52). In our studies, we observed that the virus-infected P2X7r KO mice had lower levels of proinflammatory cytokines such as IL-6, TNF-α, IFN-γ, CXCL10, and CCL2 in their lungs at day 3 postinfection. Activation of P2X7 receptors by extracellular ATP plays a central role in the release of proinflammatory cytokines, and it plays a role on the regulation of macrophages, neutrophils, and effector T cells, important cell mediators during inflammation (9, 11, 53, 54). Therefore, the lower level of cytokines observed in the lungs of the P2X7r KO mice after influenza virus infection may be explained by the lack of activation of this purinergic signaling pathway in these mice. We observed that IL-10 levels in the lungs of the KO mice infected with PR/8 or NL/09 were lower than those of the WT mice. IL-10 is considered an anti-inflammatory cytokine in the context of influenza virus infection, and its production relates to reduced immunopathology and increased survival (41). This discrepancy may be explained by the time when we measured IL-10 in the lungs, since the main contributors to IL-10 production in the context of influenza infection are CD8^+^ effector T cells, a population of cells that is found later in infection (41). In addition, increased levels of this cytokine early during infection can hinder the activation of the antiviral immune response (53), suggesting that the increased levels observed in the WT mice early during infection may be detrimental.

The suppression of cytokine production at the site of infection using drugs (55) or mice with specific cytokines knocked out (56–58) is not enough to confer full protection against lethality during influenza virus infection. The lack of the receptor in our model does not induce a global reduction in cytokine production after influenza virus infection since we did not observe differences in cytokine levels in serum from the virus-infected KO and WT mice. We also measured the basal levels of IL-1β, IL-6, TNF-α, and CCL5 in the lungs of naive KO and WT mice, and no significant differences were observed (see Fig. S2 in the supplemental material). Together, these results suggest that the reduced histopathology and increased survival observed in the KO mice after infection are in part due to a protective immune response at the site of infection rather than ablation of the response.

10.1128/mBio.00229-17.2 FIG S2  Cytokines in the lungs of naive purinergic receptor P2X7 knockout or wild-type mice. Lungs (*n* = 5) were collected on day 3 after PBS instillation and processed for multiplex ELISA to determine the amount of cytokine protein (in picograms per milliliter) in each sample. Cytokines and chemokines evaluated included interleukin-1β (IL-1β), IL-6, tumor necrosis alpha (TNF-α), and CCL5. Download FIG S2, PDF file, 0.2 MB. Copyright © 2017 Leyva-Grado et al. 2017 Leyva-Grado et al. This content is distributed under the terms of the Creative Commons Attribution 4.0 International license.

The local increase in cytokines stimulates the infiltration of neutrophils as well as monocytes from the periphery, an essential step for resolution of virus infection (2, 59). In our studies, we observed a reduction in neutrophil influx to the airways of the KO mice at day 3 postinfection that was also evident during the analysis of the lung histology sections. An exacerbated response with an increased influx of highly proinflammatory neutrophils and the prolonged activation of these cells leads to increased tissue damage and lethality (3, 6, 60). Purinergic signaling in the presence of high concentrations of extracellular ATP has a critical role in activation of neutrophils for the production of cytokines and in the increased influx and persistence of neutrophils at the site of infection (11). Furthermore, we observed a significantly smaller amount of CXCL10 in the lungs of KO mice infected with NL/09 or PR/8 influenza virus. This chemokine is an important factor for the chemotaxis and activation (increased oxidative burst) of neutrophils, and high levels in the respiratory tract after influenza virus infection correlate with enhanced pulmonary immunopathology (61–63). Together, these results suggest that purinergic signaling through the P2X7 receptor is important for the recruitment and possible activation of neutrophils in the lungs after influenza virus infection.

We observed an increased influx of activated macrophages to the airway space in the KO mice compared to the WT group. In addition, flow cytometry analysis of the lungs in virus-infected mice showed an increased number of CD11b^+^ macrophages or inflammatory macrophages in the KO mice compared to the WT mice. These cells can be proinflammatory or immunomodulatory based on the cytokine profile they produce and surface markers they express after activation (1). Lung accumulation of these inflammatory macrophages with an immunoregulatory phenotype contributes to reduce the development of immunopathology (64). Furthermore, it has been shown that maintenance of this cell population for a longer time at the site of infection correlates with protection of mice from influenza-induced immunopathology and lethality (65). The role of the P2X7 receptor in the activation of macrophages has been previously established *in vivo*, *ex vivo*, and *in vitro* (8). Activation of the P2X7r pathway is important for the release of proinflammatory cytokines, increases in cation fluxes, respiratory burst, inhibition of phagocytosis, and apoptosis in different populations of macrophages (19, 21, 66).

Our results indicate that not only the number of immune cells infiltrating the lung after infection determines the development of an exacerbated response leading to immune pathology, but and perhaps more important, is the type and activation status of the cells recruited to the site of infection. The histopathology analysis of lung sections clearly showed an increased number of infiltrating cells in the lung parenchyma of the virus-infected wild-type mice compared to the virus-infected KO mice. However, further analysis of the infiltrating cells showed that the number of CD11b^+^ macrophages (whole lung) and number of activated macrophages (BALF), both associated with protection, were higher in the virus-infected KO mice. Furthermore, activation of the P2X7 receptor can modify the protein secretion profile of stimulated macrophages depending on the activation status of the cells (proinflammatory or immunomodulatory), providing evidence that activation of this pathway may function to fine tune the activity of the heterogeneous population of macrophages observed through infection of the lungs (54).

Activation of the purinergic receptor P2X7 signaling by increased levels of extracellular ATP caused by influenza virus infection leads to an exacerbated immune response characterized by increased production of proinflammatory cytokines, induction of apoptosis, increased influx of neutrophils in the airways, and development of lung histopathology. This response likely produced an amplified influenza virus-induced immunopathology, leading to an increased morbidity and mortality in the infected mice. On the contrary, these effects were moderated in the P2X7r KO mice, resulting in a better outcome to infection.

Our studies demonstrated an important role for this receptor in the development of lung immunopathology during influenza virus infection. These results contribute to a better understanding on how the immune response may be directed to cause the immunopathology observed during influenza infection. Furthermore, understanding the role this receptor has in the activation of the immune response opens an opportunity for development of medical interventions aimed to modulate the host response toward a balanced immune response that will clear the virus with minimum lung damage.

## MATERIALS AND METHODS

### Purinergic receptor agonists and antagonists.

Adenocarcinomic human alveolar basal epithelial cells or A549 cells (ATCC, Manassas, VA) were evaluated to determine the presence of the purinergic receptor P2X7. A549 cells cultured on cover glass were fixed with 4% buffered formalin and later incubated with 5% nonfat milk for 30 min. After the blocking step, the cells were incubated with a polyclonal anti-P2X7 receptor antibody raised in rabbit (OriGene, Rockville, MD). For detection of the antibody, the cells were incubated with an anti-rabbit Alexa Fluor 488 antibody (Thermo Fisher Scientific, Waltham, MA). Mounting medium containing 4′,6′-diamidino-2-phenylindole (DAPI) (Vector Laboratories, Burlingame, CA) was added to the cells before analysis. To study the effects of purinergic receptor agonist and antagonists on virus growth, A549 cells were treated with brilliant blue G (BBG) (Sigma-Aldrich, St. Louis, MO) or 3-[1-[[(3′-nitro[1,1′-biphenyl]-4-yl)oxy]methyl]-3-(4-pyridinyl)propyl]-2,4-thiazolidinedione (AZ11645373) (AZ) (Tocris, Bristol, United Kingdom) (both BBG and AZ11645373 are P2X7 receptor antagonists) or with 2′(3′)-*O*-(4-benzoylbenzoyl)ATP trimethylammonium (BzATP) (Sigma-Aldrich, St. Louis, MO), a P2X7 receptor agonist. A549 cells were treated with 100 μM/ml of BBG or AZ11645373 or with BzATP 1 h before infection with influenza A/Puerto Rico/8/1934 H1N1 (PR/8) or influenza A/Netherlands/602/2009 H1N1pdm (NL/09) virus. After infection, the virus was removed, and medium with the respective compound was added to the cells. Supernatants were collected at 24 or 48 h postinfection. Samples were stored at −80°C until titration was performed by standard plaque assay on Madin-Darby canine kidney (MDCK) cells.

### Animals.

All research studies involving the use of animals were reviewed and approved by the Institutional Animal Care and Use Committees (IACUC) at the Icahn School of Medicine at Mount Sinai. This study was carried out in strict accordance with the recommendations in the *Guide for the Care and Use of Laboratory Animals* (67).

Female C57BL/6 or P2X7 receptor knockout mice (8 to 10 weeks old) purchased from Jackson Laboratories (Bar Harbor, ME) were used for all experiments. For virus challenges, mice were anesthetized by intraperitoneal injection of a mixture of ketamine (100 mg/kg of body weight) and xylazine (5 mg/kg) before intranasal administration of a lethal dose of influenza virus PR/8 or NL/09 in a volume of 30 μl. Animals were monitored daily for clinical signs of illness, and body weights were recorded daily for 14 days. Upon reaching 75% of initial body weight, animals were humanely euthanized.

### Lung virus titers.

On days 4 and 7 postinfection, groups of mice were euthanized, and the lungs were collected and homogenized (BeadBlaster 24; Benchmark Scientific) in 1 ml of sterile phosphate-buffered saline (PBS). The lung homogenates were spun at 16,000 × *g* for 10 min to pellet tissue debris, and the supernatants were collected. Samples were stored at −80°C until titration was performed by standard plaque assay on MDCK cells.

### Histopathology.

Mice were euthanized on day 4 or 7 postinfection, and their lungs were perfused *in situ* with 1 ml of 10% buffered formalin. Lung sections were stained with hematoxylin-eosin and evaluated by an experienced comparative pathologist from the Center of Comparative Medicine and Surgery in the Icahn School of Medicine at Mount Sinai. The following score system (on a scale of 0 to 4) was used to analyze the histopathology of the lung sections: 0, no lesions; 1, mild changes with scattered cell necrosis/vacuolation and few/scattered inflammatory cells in the bronchiolar epithelium with minimal perivascular inflammation; 2, moderate multifocal vacuolation with peribronchiolar inflammation (<5 cell layers thick); 3, marked, multifocal/segmental necrosis, epithelial loss/effacement, and peribronchiolar inflammation (>5 cell layers thick); and 4, severe, coalescing areas of necrosis, parenchymal effacement with confluent areas of inflammation. Overall pathology scores per group were used for the statistical analysis of the results.

Additional sections were processed for immunohistochemistry (IHC) and used to determine the amount of the apoptosis-related protein caspase-3. Ten-micron-thick deparaffinized sections were subjected to heat-induced epitope retrieval by boiling in 10 mM sodium citrate buffer (pH 6) for 10 min, followed by a 30 min cool-down. Cleaved caspase-3 antibody (Cell Signaling Technology, Inc., Beverly, MA) was diluted 1:1,000 in the following buffer: 0.1 M PBS, 0.3% (wt/vol) bovine serum albumin (BSA), 0.1% (wt/vol) sodium azide, 0.06% (wt/vol) *n*-ethyl-maleimide, and 20% (vol/vol) glycerol. This antibody specifically recognizes the large fragment (17/19 kDa) of the active protein (cleaved), but not full-length caspase-3. Cleaved caspase-3-positive cells were counted in printed digital images using a transparent template with a rectangular box measured 0.25 mm by 0.5 mm for photographs taken at a magnification of ×20 in three different fields. An individual blind to the experimental treatments completed the quantification.

### Bone marrow-derived macrophages.

Bone marrow-derived macrophages (BMDM) were generated by culturing total bone marrow from femurs and tibias of P2X7 receptor KO or wild-type mice as previously described (68). Collected cells were plated at a density of 1 × 10^6^ cells/well in six-well plates using high-glucose Dulbecco modified Eagle medium (DMEM) supplemented with 10% fetal bovine serum (FBS), 10 mM penicillin-streptomycin, 10 mM sodium pyruvate, 10 mM HEPES, 50 μM 2-mercaptoethanol, and 20% L929 cell conditioned supernatant containing macrophage colony-stimulating factor (M-CSF). The cells were cultured for 5 days, after which the medium and nonadherent cells were removed, and fresh medium was added every other day until harvest between days 8 and 10. The cells were purified by adherence. To harvest the cells, we used 500 µl of 0.5% trypsin. For infection studies, cells were plated in 12-well plates and infected with influenza A NL/09 virus at a multiplicity of infection (MOI) of 1 for 48 h. Supernatants of samples were collected at 24 and 48 h postinfection and analyzed for virus titer and cytokine production.

### Cytokine and chemokine quantification.

Protein levels of cytokines/chemokines were evaluated using a multiplex bead array assay (Milliplex; EMD Millipore, Billerica, MA) in cell culture supernatant of bone marrow-derived macrophages infected with influenza NL/09 or in lung homogenates and serum samples from mice infected with influenza virus NL/09 or PR/8 according to the manufacturer’s instructions. The assays were run using 1,500 beads per set of each of cytokines measured per well in a total volume of 50 µl. The plates were read on a Luminex MAGPIX platform. For each bead set, >50 beads were collected. The median fluorescence intensity of these beads was recorded and used for analysis with the Milliplex software using a 5P regression algorithm.

### Flow cytometry.

Mice were euthanized by intraperitoneal administration of pentobarbital at the indicated time points. Lungs were isolated in HBSS buffer (Sigma, St. Louis, MO), digested with type IV collagenase (Worthington Biochemical, Lakewood, NJ) for 30 min with agitation, and then processed into single-cell suspensions by forcing the digested lungs through a 70-µm nylon cell strainer (Falcon, Corning, NY). Single-cell suspensions were stained in PBS supplemented with 1% bovine serum albumin and 2 mM EDTA. Antibodies against the following immune markers were used for discrimination of immune cell types: CD45 (30-F11; BD), CD11b (M1/70; BioLegend), CD11c (HL3; BD), Ly6c (HK1.4; eBioscience), GR1 (RB6-8C5; eBioscience), major histocompatibility complex class II (MHCII) (M5/114.15.2; BD), and SiglecF (E50-2440; BD). Live cells were discriminated from dead cells by using a viability dye eFluor 520 (eBioscience). All flow cytometry data were acquired on an LSR-II flow cytometer (BD) and analyzed using FlowJo software (FlowJo LCC, Ashland, OR). Cell sorting was performed on a FACS Aria (BD).

### Bronchoalveolar lavage cytospin.

Mice were euthanized by intraperitoneal administration of pentobarbital at the indicated time points. Leukocytes recruited to the airway space were collected by lavaging the lungs *in situ* by slowly injecting 1 ml of cold sterile PBS using an input 3-ml syringe with three-way stopcock and then approximately 1 ml of bronchoalveolar lavage fluid (BALF) was collected from lungs using the output 3-ml syringe. We repeated this procedure three times. Cell number and cell viability were determined using trypan blue and an automatic cell counter (Countess II; Thermo Fisher Scientific, Waltham, MA). For the cytospin, 300 µl of each BALF sample containing 1 × 10^5^ cells was spun at 600 rpm for 10 min. The slides were air dried for 2 h and then stained with Diff-Quick. The slides were evaluated in a blind manner by a trained comparative pathologist. Differential cell counts (percentage) were established by counting at least 200 cells at a high magnification (×600) using the following categorization (69). Undifferentiated alveolar macrophages were defined as relatively large, round to oval cells with eccentric round to oval nuclei and abundant granular cytoplasm, while activated macrophages were larger and contain more cytoplasm and numerous cytoplasmic vacuoles, with some macrophages binucleated. Neutrophils (10 to 15 µm in diameter) were characterized by an eosinophilic cytoplasm and a basophilic lobulated nucleus with violet granules. Inactivated lymphocytes were small (<10 µm) cells with rounded nuclei, dense nuclear chromatin, and little cytoplasm, while activated (reactive) lymphocytes were larger with more basophilic cytoplasm and eccentric nuclei. Finally, we also quantified the percentage of lysed cells by the relative proportion of isolated (naked) nuclei.

### Statistical analysis.

Statistical differences in virus titers and cytokine production between the KO and WT mice were analyzed using Student’s *t* test analysis followed by the Holm-Sidak test. For analysis of survival rates, Kaplan-Meier curves were compared using the log rank (Mantel-Cox) test. For analysis of the flow cytometry results, we used a two-way analysis of variance followed by multiple comparisons using the Tukey posttest. Error bars indicate standard errors of the means. *P* values of <0.05 were considered significant. All the analyses were performed using the GraphPad software (La Jolla, CA).
