# Supplementary material for: Contribution of the Purinergic Receptor P2X7 to Development of Lung Immunopathology during Influenza Virus Infection
Source: mBio. 2017 Mar 28;8(2):e00229-17. doi: 10.1128/mBio.00229-17 (PMC5371412; doi:10.1128/mBio.00229-17)
Supplement: FIG S1 [file mbo002173262sf1.pdf]

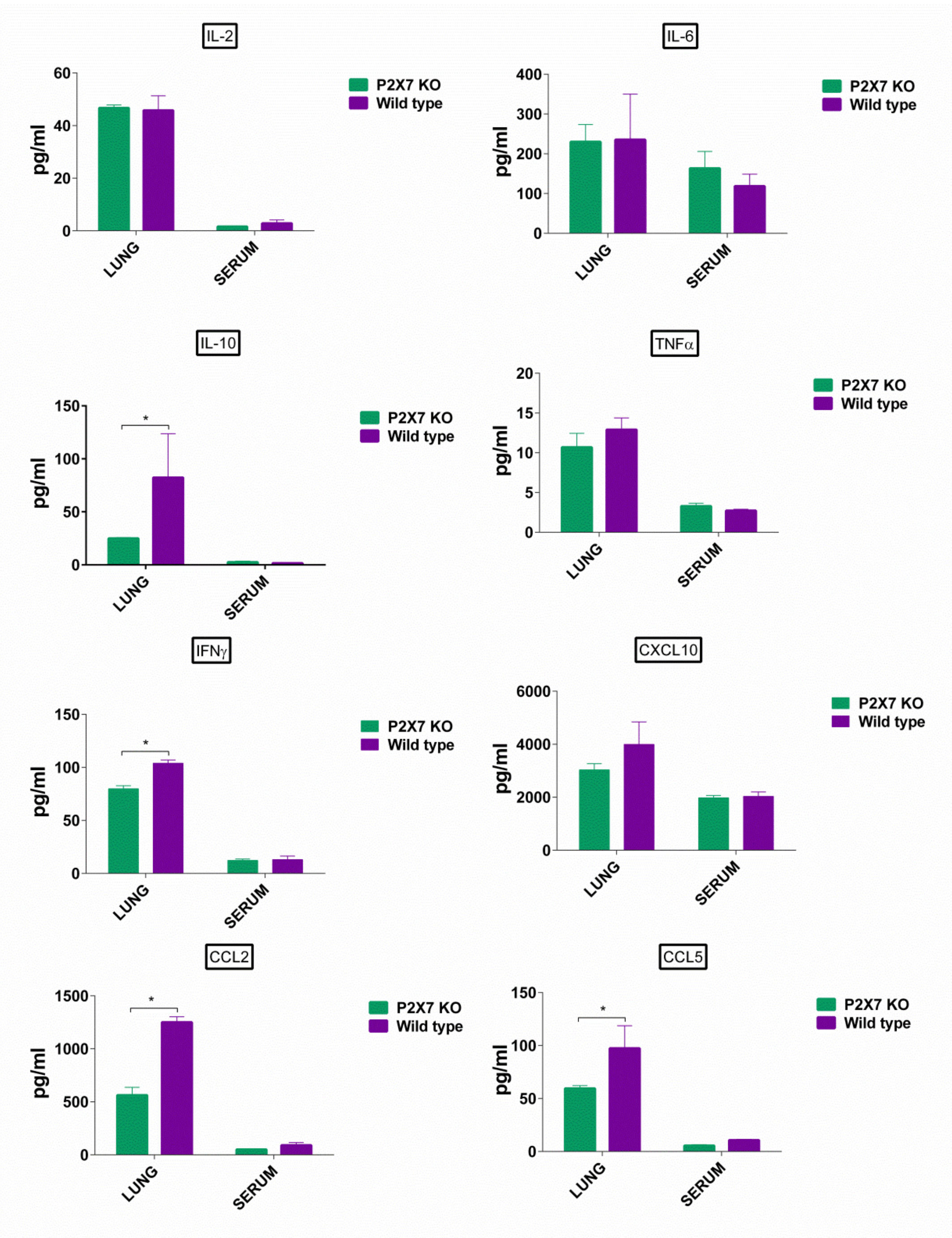

Figure S1. Lung cytokine production in purinergic receptor P2x7 knockout or wild type mice infected with influenza A/Puerto Rico/08/1934 H1N1 virus. Lungs and serum samples (n=5 per group) were collected on day 3 post-infection and processed for multiplex ELISA analysis to determine the amount of cytokine protein (pg/ml) in each sample. Cytokines and chemokines evaluated included Interleukin (IL)-2, IL-6, IL-10, Tumor necrosis alpha (TNF-α), Interferon gamma (IFN-γ), CXCL10, CCL2 and CCL5. (*) indicates a significant difference $p<0.05$.
