# Supplementary material for: Contribution of the Purinergic Receptor P2X7 to Development of Lung Immunopathology during Influenza Virus Infection
Source: mBio. 2017 Mar 28;8(2):e00229-17. doi: 10.1128/mBio.00229-17 (PMC5371412; doi:10.1128/mBio.00229-17)
Supplement: FIG S2 [file mbo002173262sf2.pdf]

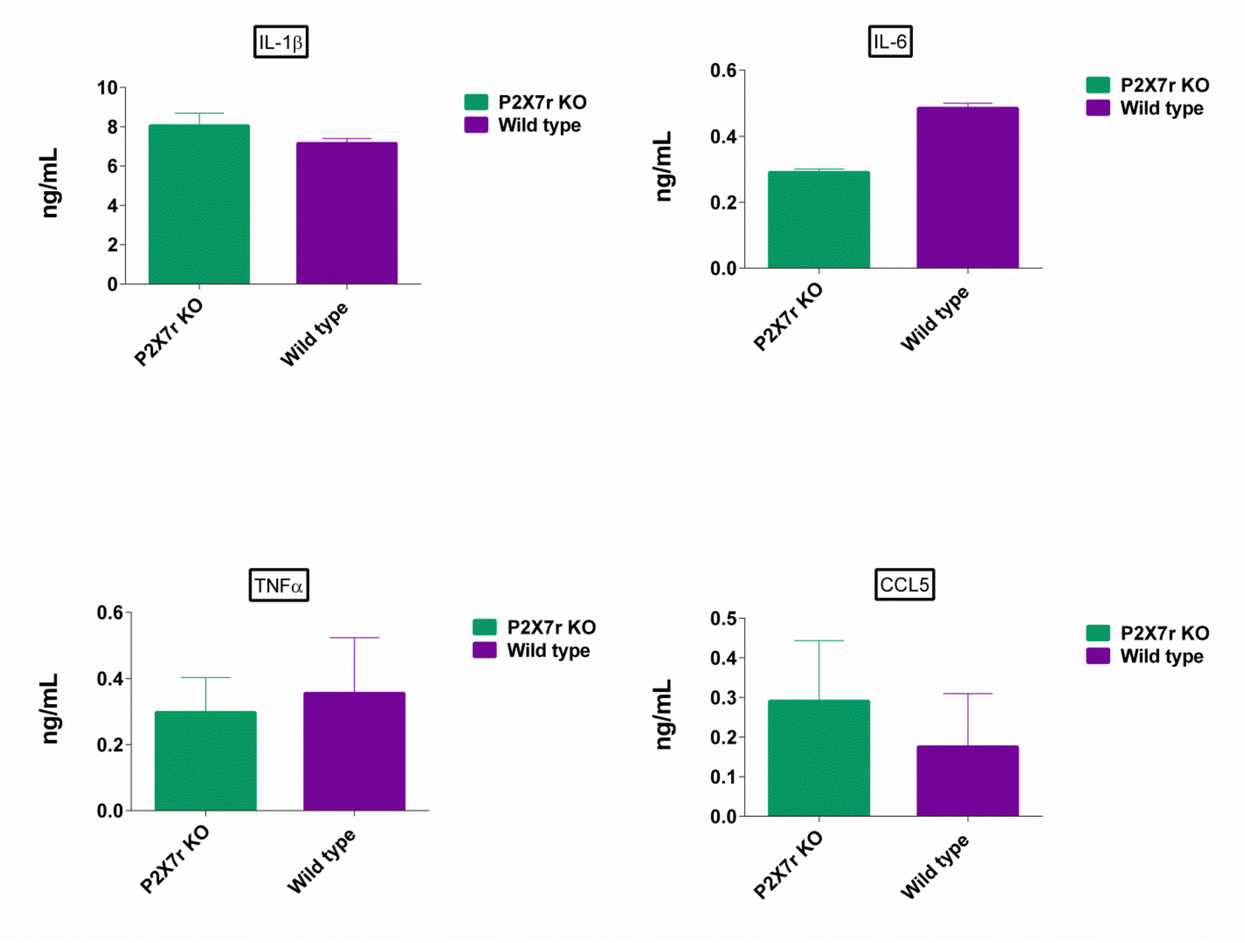

Figure S2. Lung cytokines in lungs of naïve purinergic receptor P2x7 knockout or wild type mice. Lungs were collected on day 3 after PBS instillation and processed for multiplex ELISA analysis to determine the amount of cytokine protein (pg/ml) in each sample. Cytokines and chemokines evaluated included Interleukin (IL)-1β, IL-6, Tumor necrosis alpha (TNF-α), and CCL5.
